# Student-led interprofessional global health course: learning impacts during a global crisis

**DOI:** 10.1186/s12909-023-04116-4

**Published:** 2023-03-16

**Authors:** Anne Xuan-Lan Nguyen, Lucille Xiang, Radhika Chhibber, Hailey Blanchard, Svetlana Tikhonova, Hiba Zafran, Catherine-Anne Miller, Yves Bergevin

**Affiliations:** 1 Faculty of Medicine and Health Sciences, McGill University, 3706 Peel St, H3A 1W9 Montreal, QC Canada; 2 School of Physical and Occupational Therapy, Faculty of Medicine and Health Sciences, McGill University, Montreal, QC Canada; 3 Faculty of Dental Medicine and Oral Health Sciences, McGill University, Montreal, QC Canada; 4 Ingram School of Nursing, Faculty of Medicine and Health Sciences, McGill University, Montreal, QC Canada

**Keywords:** Global health, Medical education, Learning, Interprofessionalism, Student initiative

## Abstract

**Background:**

This study assesses the impact of the Interprofessional Global Health Course (IPGHC) on students’ fundamental global health knowledge and personal viewpoints on global health domains. It explores the evolution of students’ understanding of global health specifically in relation to the COVID-19 pandemic.

**Methods:**

Ninety-nine students were selected from 123 McGill student applicants based on their motivation and commitment to take part in IPGHC’s ten-week 2020 curriculum. These IPGHC students were eligible to participate in the study. The study’s design is sequential explanatory mixed methods. The cross-sectional survey (quantitative phase) appraises students’ global health learning outcomes using pre- and post-course surveys, with the use of 5-point Likert-scale questions. The descriptive qualitative survey (qualitative phase) further explores the impact of IPGHC on student’s understanding of global health and the reflections of students on the COVID-19 pandemic after IPGHC. The post-course survey included a course evaluation for quality improvement purposes.

**Results:**

Of the 99 students, 81 students across multiple undergraduate and graduate disciplines participated in the study by completing the course surveys. Mean knowledge scores of the following 11 global health topics were increased between pre- and post-course survey: Canadian Indigenous health (*P* < 0.001), global burden of disease (*P* < 0.001), global surgery (*P* < 0.001), infectious diseases and neglected tropical diseases (*P* < 0.001), refugee and immigrant health (*P* < 0.001), research and development of drugs (*P* < 0.001), role of politics and policies in global health (*P* = 0.02), role of technology in global health (*P* < 0.001), sexual violence (*P* < 0.001), systemic racism in healthcare (*P* = 0.03), and trauma in the global health context (*P* < 0.001). A positive change in student viewpoints was observed in response to questions regarding their perception of the importance of global health education in their own professional health care programs (*P* < 0.001), and their understanding of the roles and responsibilities of other healthcare professionals (*P* < 0.001). In the post-course survey open-ended questions, students exemplified their knowledge gained during the course to create a more informed definition of global health. Several recurring themes were identified in the student reflections on the COVID-19 pandemic, notably policy and politics, followed by access to healthcare and resources.

**Conclusion:**

This study emphasizes the need for interprofessional global health education at the university level and demonstrates how rapidly global health learners can apply their knowledge to evolving contexts like the COVID-19 pandemic.

**Supplementary Information:**

The online version contains supplementary material available at 10.1186/s12909-023-04116-4.

## Background

During the past decade, the field of global health has undergone major changes: more healthcare professionals are getting involved in global health projects and universities are creating majors centered around its principles and science [1]. Students are increasingly exploring ways to get involved in global health through research projects, local and international community service internships, study abroad programs, and other initiatives [2]. With the COVID-19 pandemic, interest in infectious diseases and public health research has amplified [3]. The pandemic has exacerbated existing and widening gaps in health equity [4]. To address global health in an era grappling with colonial structures and violence, academics and programs must emphasize culturally-safe and ethically-aware training and practices prior to the global health experience to ensure students do reflexively mitigate harms within local communities [5].

While global health actions often involve multiple stakeholders, preparation and training for educational global health experiences have not always incorporated the interprofessional nature of the field [6]. To prepare students for clinical, research or program-based practice in global health, it is important to introduce them to interprofessionalism and the roles of the different professionals that make up an interprofessional team [7]. Collaborative and coordinated efforts amongst many disciplines and professions are necessary to respond to global health crises [8]. Interprofessional health education has further shown to improve patient care [9]. Widely utilised by universities, the National Interprofessional Competency Framework is a model for educating health professionals and is grounded on four key principles: role clarification, interprofessional conflict resolution, team functioning, and collaborative leadership [10]. While these domains are utilised nationally in healthcare settings, interprofessional education is recognised by the World Health Organization (WHO) in a broader sense as necessary to address global health crises and disparities [11]. This has further shaped the development of global health programs among both the undergraduate and graduate levels in recent years. Several related trends are also on the rise: a greater student awareness and interest in global problems, increased demand for educational possibilities to meet this interest, heightened public awareness of the global health agenda and expansion of public and private funding for international health initiatives [12].

The Interprofessional Global Health Course (IPGHC) is a student-led initiative at McGill University formed in 2007 to address the paucity of Global Health curricula and foster student leadership in global health. IPGHC recognizes the important role students have in the future of global health governance, clinical practice, and global health interactions in their respective countries. Every year, the course has four student coordinators and a faculty advisory committee from four healthcare disciplines at McGill University who manage all aspects of the course ranging from selection and enrollment of students into the course, to curriculum design and implementation. Students are also tasked to create of interactive cases and review assignments. To our knowledge, no study has evaluated the perception of students from diverse healthcare disciplines enrolled in an interprofessional global health course amid the pandemic that focused on developing fundamental global health knowledge and competencies [13]. The objectives of this study were to assess how the IPGHC course changed students’ perception of their knowledge in core global health domains and to explore students’ understanding of global health specifically in relation to the COVID-19 pandemic.

## Methods

### Study Design

We applied a sequential explanatory mixed methods design [14]. The study is composed of a cross-sectional survey (quantitative phase) to appraise the learning outcomes of students in the IPGHC course on global health competencies, and a descriptive qualitative survey (qualitative phase) to explore key quantitative findings in more depth. The authors further assessed how knowledge gained in IPGHC informed students’ understanding of the emergence of the COVID-19 crisis.

### Intervention: Interprofessional Global Health Course

The IPGHC is a free student-led course consisting of a ten-week curriculum including experts’ presentations and interactive interprofessional activities on different global health themes, inspired by the Sustainable Development Goals (SDGs), each week (Table 1). To receive co-curricular credit for the course, students were required to be present in at least 80% of classes, to complete pre- and post-course surveys and write a final reflective essay examining the COVID-19 pandemic through a lens from the course (Appendices 1–2). The entire course took place from January 7th, 2020 (first workshop) to May 15th, 2020 (deadline for the final assignment).

**Table 1:** Topics covered through the course

Week: Date	Topic	Global Health Speaker Professional Field	Sustainable Development Goals (SDGs) Focus
Week 1: January 7, 2020	Introduction to global health	Medicine	All SDGs
Week 2: January 14, 2020	Reproductive, maternal and child health: scaling-up for sustainable impact	Medicine	Quality education (#4), gender equality (#5), zero hunger (#2), reduce inequalities (#10)
Week 3: January 21, 2020	Indigenous health	Nursing	All SDGs
Week 4: January 28	Lancet: oral health as global health	Dentistry	All SDGs
Week 5: February 4, 2020	Contemporary issues in humanitarian action	Nursing	No poverty (#1), partnerships for the goals (#17)
Week 6: February 11, 2020	Mental health in global health	Psychiatry	Good health and well-being (#3)
Week 7: February 18, 2020	Ecosystems approach to health and climate	Geography	Responsible consumption and production (#12), climate action (#13), life below water (#14), life on land (#15)
Week 8: February 25, 2020	Politics and policy	Physical and occupational therapy	Decent work and economic growth (#8), industry, innovation, and infrastructure (#9), peace, justice, and strong institutions (#16)
*Formal global declaration of the pandemic (March 11, 2020)*			
Week 9: March 17, 2020	Water, sanitation, hygiene (online)	Engineering	Clean water and sanitation (#6), affordable and clean energy (#7)

### Sample: eligibility criteria

Out of the 123 students who submitted a registration form to the course, 99 undergraduate and graduate students from over 20 disciplines at McGill University were enrolled in the IPGHC. The course was capped at the maximum occupancy of the teaching room (< 100 students). Students were selected to the course based on their year of study, motivations for taking the IPGHC, previous experience in global health, their commitment to attend lectures and to complete the final assignment. Of note, 20 student spots were reserved for each of the four core healthcare professional schools (nursing, medicine, dentistry, and physiotherapy-occupational therapy) (Table 2).

**Table 2:** Descriptive statistics of student sample

Sample characteristics	Number of students (%)
Medical students	17
Nursing students	13
Dental students	9
Physical and occupational therapy (PT/OT) students	10
Other*	31
Post-graduate students	1

*= Law, international development, biomedical sciences, dietetics, anatomy and cell biology, microbiology and immunology, anthropology, nutritional science

### Data Collection

The pre- and post-course surveys (Appendices 1, 2) included questions about participant demographics (email, participant code, program, and year of study), as well as multiple choice and open-ended questions about participants’ global health understanding, experience, and skills. Pre-course surveys were completed in December 2019 (two weeks prior to the course), while post-course surveys were completed in May 2020 (within two months following the course). Participants were asked to rate a set of statements regarding their personal viewpoints on a 5-point Likert-scale (1 = strongly disagree, 2 = somewhat disagree, 3 = neutral, 4 = somewhat agree, 5 = strongly agree). Another set of statements regarding their knowledge of global health topics was also rated on a 5-point Likert-scale (1 = very poor, 2 = poor, 3 = fair, 4 = good, 5 = very good). The mean score and standard deviation for each question was recorded. The post-course survey contained the pre-course survey questions (Appendix 2 A) and course evaluation portion for quality improvement purposes (Appendix 2B). Students further responded to this prompt: “Using a lens from the McGill IPGHC, analyze the COVID-19 pandemic” in a two-page double-spaced reflective essay. To encourage students to provide feedback on the course, students were required to complete all surveys to receive a certificate of completion for the course. Data was aggregated into pre-course and post-course to understand trends in knowledge gained.

### Data Analysis

#### Pre-course and post-course surveys

Descriptive statistics regarding characteristics of the survey sample were performed. Continuous variables were expressed as means, and categorical variables were expressed as proportions. Student paired sample t-tests were used to examine the statistical significance of the change between pre and post course surveys for the same sample group. RStudio (version 1.1.456) was used to analyse the data. A p-value ≤ 0.05 was taken as the criterion for statistical significance.

#### Final reflective essay

The student course-coordinators analysed the reflective essays to understand the impact of the course on their perception of global health. Reflections were grouped by answer and read by all four coordinators. The team conducted a thematic analysis of the data to understand how the IPGH course has impacted students’ perspectives. The approach to thematic analysis was informed by guidelines and principles in grounded theory [15]. In this study, the strategy consisted of three main stages: initial coding, focused coding, and theoretical coding. In the first stage, all four student coordinators remained open to all possible emergent themes indicated by readings of the data. On the other hand, focused coding involves the inductive categorization of data based on thematic similarity at the level of description. Finally, theoretical coding integrates thematic categories into core theoretical constructs at a higher level of analysis [12]. At this stage, all four student coordinators labelled the essays with different global health themes. If three or more student coordinators identified the same theme to an essay, this theme was attributed to the essay. The thematic analysis was performed on the reflective essays using Atlas.ti (version 9.0.1).

### Study approval

This study was granted ethical approval by the University of McGill Faculty of Medicine and Health Sciences Institutional Review Board l (#A12-E99-09B). All methods were performed in accordance with the Declarations of Helsinki.

## Results

### Demographics by profession

Of 99 students registered for the course, 81 attended at least 8 out of 10 classes, completed the pre- and post-course surveys, and wrote the final reflection. The sample consisted of medical students (17%), nursing students (13%), physical and occupational therapy students (10%), dental students (9%), post-graduate students (1%), and the remaining students (31%) were from other fields (law, international development, biomedical sciences, dietetics, anatomy and cell biology, microbiology and immunology, anthropology, nutritional science) (Table 2).

### Pre-course and post-course surveys

#### Global health themes (quantitative results)

**Fig. 1:**
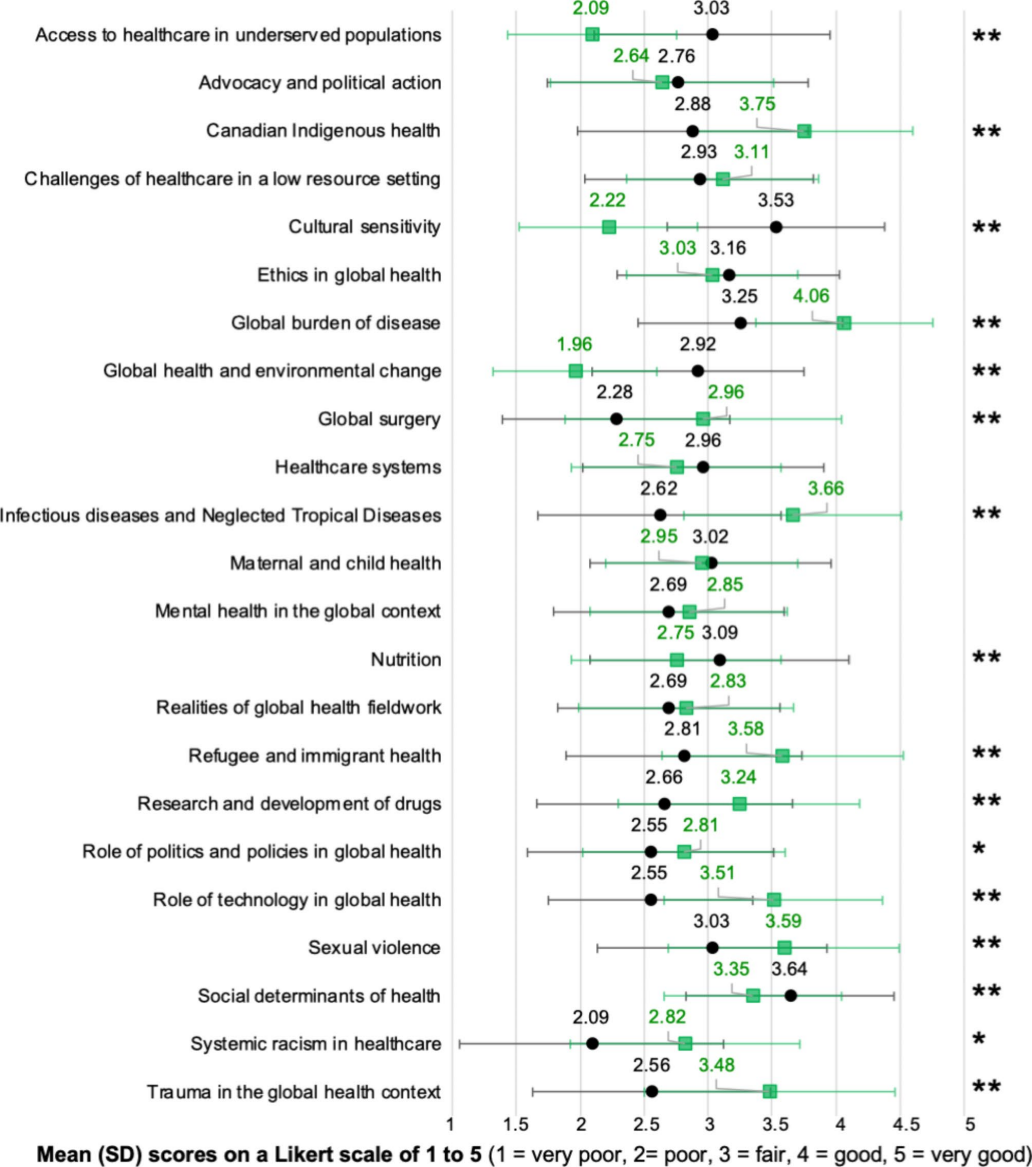
Students’ ratings of their knowledge of global health topics before and after the course, on a Likert scale of 1 to 5 (1 = very poor, 2 = poor, 3 = fair, 4 = good, 5 = very good) Legend: The black circles and lines represent the pre-course survey responses’ means and standard deviation, respectively. The green squares and lines represent the post-course survey responses’ means and standard deviation, respectively. The asterisk refers to a p-value of less than 0.05. The double asterisk refers to a p-value of less than 0.001

Positive change in student’s knowledge were observed with regards to eleven global health topics: Canadian Indigenous health (*P* < 0.001), global burden of disease (*P* < 0.001), global surgery (*P* < 0.001), infectious diseases and neglected tropical diseases (*P* < 0.001), refugee and immigrant health (*P* < 0.001), research and development of drugs (*P* < 0.001), role of politics and policies in global health (*P* = 0.02), role of technology in global health (*P* < 0.001), sexual violence (*P* < 0.001), systemic racism in healthcare (*P* = 0.03), and trauma in the global health context (*P* < 0.001). There was a decrease in students’ knowledge of five global health topics: access to healthcare in underserved populations (*P* < 0.001), cultural sensitivity (*P* < 0.001), global health and environmental change (*P* < 0.001), nutrition (*P* < 0.001), and social determinants of health (*P* < 0.001). Ratings to the seven other global health topics did not report a significant difference between the pre- and post-course surveys (Fig. 1).

#### Personal viewpoints (quantitative results)

**Fig. 2:**
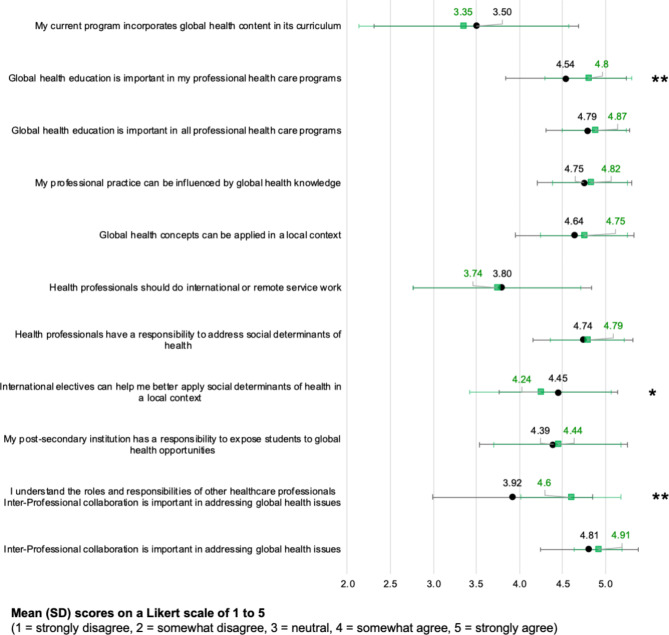
Students’ ratings of their personal viewpoints before and after the course, on a Likert scale of 1 to 5 (1 = strongly disagree, 2 = somewhat disagree, 3 = neutral, 4 = somewhat agree, 5 = strongly agree) Legend: The black circles and lines represent the pre-course survey responses’ means and standard deviation, respectively. The green squares and lines represent the post-course survey responses’ means and standard deviation, respectively. The asterisk refers to a p-value of less than 0.05. The double asterisk refers to a p-value of less than 0.001

A significantly positive change in student viewpoints was observed in response to questions regarding their perception of the importance of global health education in their own professional healthcare programs (*P* < 0.001), and their understanding of the roles and responsibilities of other healthcare professionals (*P* < 0.001). There was a decrease in students’ ratings of the application of social determinants of health in a local context through international electives (*P* = 0.04). Ratings to all other questions regarding their personal viewpoints did not report a significant difference between the pre- and post-course surveys (Fig. 2).

#### Understanding of “Global Health” before and after the course (qualitative results)

Pre-course, most students defined global health broadly while using the following terms: “Health for all in the world”, “the level and status of health in the world as the common context for all people”. Others specifically focused on disease control: “[Global health] is the collection of diseases or infections, such as HIV, AIDS, malaria, etc., that are impacting the world globally and how healthcare professionals from around the world can work together to treat and irradiate these illnesses.” Some students evoked more advanced concepts related to equity, cultural sensitivity and how health is interdependent with policies, economic, environmental, and social factors. For example, one student defines global health as “a discipline that studies the differences in access to healthcare, how diverse environments and socioeconomic factors can shape one’s exposure to certain diseases and their access to treatment”, and another further highlights that global health ensures that healthcare needs of every individual on the planet are met in an accessible and especially culturally sensitive manner.

Post-course, most students provided more detailed answers than in the pre-course survey while using focused phrases such as: “the study of inequalities of healthcare and the access to proper health that exist both locally and globally due to power relationships, inequalities and differences in gender and ethnic groups”. These responses evoked a more informed definition of global health through an equitable and culturally safe lens. Students further connected the concept of global health to globalization, and highlighted how economic, environmental and social processes affect people’s health in their updated definition of global health. By the end of the course, some refined definitions of global health were directly linked the COVID-19 global health crisis like the following:

We are currently in the midst of a global health crisis, and it’s very apparent now more than ever the health disparities between nations. Global Health is a term that encompasses the access to healthcare globally and the opportunity for nations to help other nations and individuals to help their worldwide brothers and sisters either by direct contributions of time or money or through advocacy. There are a number of global health goals that we are working towards, and it basically is working towards a future where there is more equity between nations in terms of healthcare.

### Final reflective essay (qualitative results)

Fourteen recurring themes were identified in the student reflections on the COVID-19 pandemic (Table 3), the top three being policy and politics (elicited in 69 essays), followed by access to healthcare and resources (52 essays) and maternal health (43 essays). Sample responses and their inferences are presented in Table 3. These sample quotes detail participants’ views of the pandemic: participants notably highlight how the pandemic has influenced the vulnerabilities of community stakeholders, and its alarming impact in achieving the SDGs.

**Table 3:** Emergent themes found in the reflective essays, accompanied by the number of essays attributed to these themes and samples of essay responses related to the themes

Themes	# of essays	Samples of essay responses	Inference
Policy, Politics and Ethics	69	“I am reminded of the importance of government legitimacy. The population needs to trust the government and public health decisions. This trust had to be built in the years before the disaster by having a strong civil society, allowing the growth of social capital, and from the government taking action to ensure population well-being.”	Within this theme, students presented ideas around rapid and coordinated governmental action in responding to pandemics, the role of inter-governmental agencies like the WHO, and ways to improve international models for care. Other students reflected on the various actors involved in a public health response: *“government, economic, sanitation, climate, politicians”.* In doing so, it was clear students who chose to reflect on this topic understood the roles of policy and politics as well as the role of public trust in government institutions may have on a response to a public health crisis such as COVID-19.
Access to Healthcare and Resources	52	“Many inequalities have become increasingly apparent depending on the demographics of a certain region, the extent to which reliable information is available and the government’s efficiency in providing the appropriate protective equipment to health professionals and other citizens.”	Most of the submitted reflections discussed inequalities and disparities in access to healthcare resources exacerbated by COVID-19.
Maternal Health	43	“The COVID-19 pandemic also has an impact on maternal and child health. Indeed, delivery procedures are impacted by the crisis. In Canada some mothers now must deliver their babies in the absence of their significant other in an effort to reduce the number of people visiting hospitals. In some part of the world, it also means that preventable birth-related complications may be fatal due to a lack of resources.”	Many reflections also highlighted the impact of the pandemic on maternal and child health, emphasizing the lack of support due to hospital capacity restrictions and a strain on resources due to the pandemic.
Elderly	27	“Covid-19 raised awareness of these issues in a broken system for our elderly population.”	Several reflections also noted the gap in care for the geriatric population.
Indigenous Health	23	“Indigenous communities across the country where access to clean water and territorial rights were already important issues are now bracing the challenges of ensuring food, water and medicine security in a period where baseline services are slowing down everywhere.” “The indigenous communities are also affected by the COVID-19. In Canada, the government decided to help these communities by supporting them at both the individual level and the organizational/community level since they are prone to face many barriers to access healthcare services. Thus, special funds were created and some payments have been deferred.”	Students noted that the challenges of food, water and health insecurity faced by Indigenous communities were worsened by the pandemic.
Racial/Ethnic Disparities	21	“Stigmatization and racism towards those who are assumed to be from China has increased exponentially. People’s fight or flight instincts kick in, resulting in knee-jerk reactions that are extremely selfish and individualistic.” “The effects of the pandemic on mental health are massive. Speaking to the general populace – it is unnatural to stay inside for so long – we miss touch, conversations with others, and traditional social settings. Religions may no longer be practiced in the same way (for example: Ramadan). Jobs are terminated or relegated to online, reducing our ability to fulfill the human need for connection. Many able-bodied healthy people have been able to overcome these issues through applications like Zoom and going for walks outside.”	Within this theme, students highlighted increased discrimination towards East Asian populations as a result of the pandemic and the impact on the public’s ability to practice religion.
Climate Change	18	“Finally, this pandemic may bring some positives aspects to our world. First, it seems like the outside world can finally take a breath of nice fresh air. Indeed, since fewer cars are outside, the level of CO2 have dropped considerably. This is good news, especially for China who has been struggling with polluted air for years. More locally, Montreal has also decreased its level of smog, and we are finally able to see Montreal a little more clearly when we are on top of the Mont-Royal Mountain.”	Students emphasized the positive impact of the pandemic on environmental health in relation to air quality both locally and abroad.
Poverty	16	“People who have low financial stability have increased barriers to getting access and surviving this pandemic. In addition to access to medical services, the shortages of food, the loss of jobs, the forced isolations and the panic buying, all put already vulnerable groups in extremely difficult situations.” “it cuts the finances of people who live paycheck to paycheck because of the lack of resources, it affects elders who are left alone in their homes or residents, unable to see their families or contest improper care. It leaves single moms struggling to find places to leave their children so that they can work to put food on the table.” “Social determinants of health, like gender and SES, help define what groups are more at risk of being negatively affected by the virus.”	Several reflections noted the increased strain for those experiencing poverty in attending to their basic needs due to the pandemic, emphasizing the importance of addressing social determinants of health.
Reproductive, Maternal and Child Health	14	“Children’s nutrition and development are also impacted by the crisis as it poses challenges for food accessibility in some regions of the world and even here in Quebec where there are accounts of desperate parents being unable to get a hold of the baby formula that their child needs. Because children are confined at home, their development may also be impacted since they are deprived from the stimulation that they receive at school and that may be lacking in certain home environments.” “Namely, women and children in the global south, indigenous communities, and the elderly, who were already at high risk or suffering from malnutrition, now face higher vulnerability.” “In many countries, the accessibility of health care is not very accessible to most women. They might not even have time to go to the hospital or a clinic to take care of themselves as they either live too far away, have to take care of the children instead of themselves, don’t have the money to afford treatment, or are uninformed. This is why, during this pandemic, health care measures must take into consideration women’s health and make treatments more accessible for them and their families.”	Students highlighted the impact of the pandemic on women and their access to health services as well as the impact of the stay-at-home order on the development of children.
Water Sanitation and Hygiene	13	“As was mentioned in the Global Health Water Sanitation and Hygiene recorded class, what to do when lacking masks or if it is acceptable to re use gloves are questions that are always present in low income countries given that lack of resources is a common problem; however, now we found ourselves having those same issues.” “Further, the lack of access to water, particularly in very remote regions, results in poor ability to perform proper hand hygiene.”	Several students linked the lack of healthcare equipment and decreased access to natural resources caused by the pandemic as a potential factor in decreased sanitation and hygiene practices, locally and abroad.
Migrants, Refugees, Asylum Seekers	12	“Populations facing poverty also feature refugees, homeless people, immigrants and migrant workers who need to keep moving and travelling to sustain their livelihoods.” “The refugees/asylum seeker demographic are one of the most prevalent marginalized population suffering from this pandemic. This is secondary to their asylum seeker status, which forces them to find low paying work despite the many comorbidities that may contribute to their deteriorating health. Additionally, due to the limited access to healthcare, they are at risk of developing serious complications from COVID as they do not have access to family doctors or primary care nurse practitioners. There is an unequal resource allocation issue that is currently placing these marginalized individuals into the direct hands of death. Why should they suffer if they are unable to describe symptoms due to language barriers, or experience cultural incompetence from the healthcare provider? Secondly, this vulnerable population have been experiencing the many financial instabilities with the downturn of the economy. Hence, the downstream effects include limited access to the appropriate protective equipment that is recommended by *Santé Publique*. If these already marginalized individual[s] can barely feed their families, how can they ensure their safety as well?”	Within this theme, several students reflected on the decrease in safety and security of the migrant, refugee and asylum seeker population as a result of the decreased access to care and services due to the pandemic.
Housing	10	“For example, the difficult housing situation and overcrowding makes it nearly impossible for the confirmed or suspected infected patients from isolating from other members of their family.”	Some students noted overcrowded housing as a barrier to following COVID-19 quarantine guidelines.
Oral Health	7	“Another concern during this class was worldwide oral health. Currently, due to the accumulation of sick people in the world, and the lack of resources to treat them, many hospitals have had to prioritize their health care services. Which makes oral care even less accessible to many communities, which is worrisome because it is deemed that 3.5 billion people globally are affected by oral conditions.”	Several reflections noted the re-prioritization of health care services, especially the decrease in access to oral care as a result of the pandemic.
Immunocompromised	6	“While I felt somewhat helpless being immunocompromised in a global pandemic, I had never experienced a helplessness to this degree. Watching and waiting as my disease flared and the government swore that all non-COVID patients who needed the meds were getting them. While I can understand the struggles of allocating a scarce resource, I feel as though the government failed to put into place the appropriate safeguards which would ensure all those who had no other treatment options would get the medication. With how helpless I felt, I can only imagine how other, even more vulnerable populations must have felt.” “Throughout my time in the global health course, I have seen time and time again how important equity is, rather than equality. For me, the COVID19 pandemic has emphasized this differentiation is as vulnerable populations tend to be forgotten and pay the price.”	Students reflected on the challenges faced by vulnerable populations including the immunocompromised, observing that global health is present for all of us.

#### Post-course evaluation

Out of the 81 students, 53 (65.4%) students did not have previous experience working or volunteering with local equity-seeking communities. The course increased the desire of 49 (60.5%) students to participate in such activities. When presented with the following statement: “the course was interesting and provided a great introduction to various global health topics”, 49 (60.5%) students strongly agreed, while 29 (35.8%) agreed, and 3 (3.7%) were neutral. 41 (50.6%) students strongly agreed, and 37 (45.7%) agreed that the course was effective in conveying the on-going global health issues around the world, while 3 (3.7%) were neutral about this statement. When presented with the final statement: “you would recommend the course to a fellow college”, 48 (59.3%) strongly agreed, 26 (32.1%) agreed, 5 (6.2%) were neutral, and 2 (2.5%) disagreed.

## Discussion

The 2020 iteration of the McGill IPGHC presented a unique opportunity to understand the importance and effectiveness of interprofessional global health education as the course took place during the onset of the COVID-19 pandemic. The interprofessional nature of this course is reflected in the course participants, which consisted of university students from a wide range of fields. IPGHC participants were medical students, nursing students, physical and occupational therapy students, dental students, but also undergraduate and graduate students majoring in other science and humanities fields. It is also important the note that the course’s interprofessionalism is also carefully planned by the selection of global health speakers. These local and international experts come from a variety of professions, ranging from medical doctors to nurses, occupational therapists, public health engineers and much more. There was an increase in students’ perception of the importance of global health in their own profession. There was also an increase in students’ understanding of the roles and responsibilities of other healthcare professionals, indicating success in the IPGHC’s goal of establishing interprofessionalism. This is regarded as one of the overall most positive outcomes for the course, as the field of global health grounded in interprofessionalism [5, 8, 9, 11]. The COVID-19 pandemic has further highlighted the need for interprofessionalism, as the global response was initiated by professionals from many fields and backgrounds [8]. As students prepare for careers in global health and the medical field, it is important they value interprofessionalism to develop collaborative and comprehensive programs for communities locally and abroad [9, 23].

Other key findings from the quantitative portion of the survey included a significant increase in knowledge of most global health topics presented, ranging from population-specific health (Indigenous health and refugee health) to global health fields (surgery, microbiology, pharmacology, policy, and technology), and structural discrimination (racism, sexism, and trauma) after taking the course. However, for certain broader topics (access to healthcare in underserved populations, cultural sensitivity, environmental change, nutrition, and social determinants of health), some students may realize that their initial perception of their knowledge on these topics did not correspond to the actual depth of the field after taking part in the lectures. This phenomenon is reported in the literature as the Dunning-Kruger Effect, which suggests that people have cognitive biases leading them to overestimate their own knowledge base [24]. By identifying their lack of knowledge in certain areas, this might have fostered them to palliate these gaps after the course.

The reflections showed the clear gain of knowledge by IPGHC students through their synthesis of information learned in the course and the application of this knowledge to understand the COVID-19 pandemic. Students’ ability to reflect on a wide-range of global health topics and their relation to the COVID-19 pandemic showed the thorough introduction to global health the IPGHC provided them with. Through the participants’ definition of global health in the post-course survey, it is evident that they recognize the relevance of mitigating COVID-19 through global health efforts. Furthermore, the answers exemplified their thinking about various populations impacted by the pandemic and the already present disparities exacerbated by the heterogeneous global responses to the coronavirus. Students demonstrated a strong interest in the policy, politics, and ethics of the pandemic, indicating this may have been a strong subject matter for the course. Global health courses in high income countries are designed to make students aware of the realities of life as a refugee or immigrant from a low-income country to address the gap between healthcare systems around the world. For many students, this course enabled them to realize that global health extends beyond low-resource settings and that global health is also relevant in a high-resource country like Canada, as evidenced in their reflections on the subject matters of Indigenous health, elderly/aging population, immunocompromised groups, and it stimulated reflections on their own positionality.

The purpose of global health education is to teach students about the effectiveness of interventions and policies that aim to improve global health, while promoting a healthy discussion of the goals of global health themselves [16]. Students demonstrated a clear increase in depth and specificity of understanding the complex domain of global health by the end of the IPGHC through the pre- and post-course surveys, becoming more in-depth and focused on equity and the future of the field with regards to globalization and the climate crisis. Analysis of these pre-course surveys provided an understanding of the rich discussions that would follow, as it was clear students had various levels of comprehension and previous experience with global health. Furthermore, it allowed the students to reflect on their initial thoughts and understandings of global health and how those would change throughout the course. The surveys further gave a quantitative measure of insight gained in the course, there was significant change in several measures indicating students’ self-reported increase in knowledge of global health.

The reflections provide a real-time example of the value of courses like IPGHC and their impact for students. The COVID-19 pandemic has shown the impacts of globalization and the role the global health field will play in combating them. An introductory, student-led, co-created and dynamically iterative course like IPGHC can enhance students’ learning on these topics and provide a space for discussion and reflection. In designing the course of global health, it is fundamental to creating and cultivating learning of worldwide health systems while bringing together students from diverse backgrounds together with complementary skills providing them with an opportunity to build shared research capacity in an ability for future global health leaders to create a future capable of scientific, clinical, healthcare and policy leaders.

### Recommendations

Globalization and the interconnectedness of the people on Earth makes health risks like the SARS-CoV-2 virus a threat to all, with capabilities to disable health systems and economies of even the most developed countries. Several insights and recommendations are suggested for future editions and models of an interprofessional global health course.

Interprofessional courses should prioritize dialogue between students from different professions, well-beyond healthcare, and create scenarios where students can actively learn together to promote collaboration and reduce professional stereotypes among healthcare students [17]. Experts from the Interprofessional Education Collaborative proposed a framework model for interprofessional collaborative practice around four core competencies including the creation of an environment of mutual respect and shared values, the recognition of professional responsibilities and roles, the promotion of interprofessional communication as well as teamwork to promote a team approach to healthcare delivery [18].

Due to the COVID-19 pandemic restrictions, the utilization of the digital platform should be maximised while ensuring the accessibility of the course content to students. Digital collaborative learning platforms have been shown to be suitable for teaching interprofessional competencies, since they facilitate social and professional exchange among learners and they can involve students experts across the globe [17]. Didactic approaches to facilitate knowledge transfer and interprofessional digital collaboration include combining the use of videos for declarative and procedural knowledge, and the use of live and synchronous interaction possibilities via virtual meetings, networking events, and online discussion forums [17].

Specifically for global health courses, there is an opportunity to recruit speakers and students from across the globe and a chance to explore and establish sustainable international educational partnerships between institutions around the world. An emphasis should be placed on inviting speakers and experts from outside the Global North to include as many perspectives as possible when it comes to equity, climate change and other global health issues.

With the establishment of meaningful educational partnerships between institutions from the Global South and Global North, students should be encouraged to reflect on the role they each may play in the global health field in general, as well as evaluate how they can empower, elevate, and collaborate with individuals from partner countries to advance health equity as a whole. Students should also reflect on how their concern for social justice, health equity and human rights are undermined by unconscious biases and by hierarchical institutions, structures and systems, which may influence their ways of thinking about global health [19].

A Nicaragua global health course elective demonstrated that global health experiences were an under-utilised opportunity to facilitate the learning of interprofessional education competencies [20]. They highlighted the role of these experiences in promoting an understanding of other professional’s roles and their relative strengths, taking down the conventional power dynamics between clinicians and fostering cultural sensitivity [20]. Student collaboration was enhanced when common goals of ‘cultural competence, patient education and problem-solving’ were emphasised throughout the course [20].

Interprofessional global health courses can be used as a platform to stimulate interprofessional problem-solving to global health issues such as the pandemic, climate crisis, power and privilege, representation of the Global South and minorities, and inequities [21, 22].

### Limitations

These findings are interpreted in the context of the study’s limitations. The survey design relied on self-assessment ratings of improvement as an evaluation tool [21]. This can be associated with a response bias, as students who had a positive experience with the course may be more likely to complete the post-course survey. Additionally, the self-assessment of learning outcomes was measured just after the course in a single site, on a rather heterogenous small subgroup of students. Indeed, the analysis of outcomes is limited by the sample size, capped by the IPGHC’s limit of 100 students per year due to logistical and financial difficulties of coordinating a free international elective. The unequal distribution of professions represented may have limited the number of interactions that a student could have with another student of a different professional track. Furthermore, the data could have been disaggregated by prior global health experience. This would have given more insight into the introductory nature of the course, as those with no initial knowledge could be compared to those with prior experience.

Additionally, while the results demonstrate value of the IPGHC in the immediate post-course period (concurring with the onset of COVID-19), they do not inform on longer-term impacts of what was learned. Future avenues include exploring the long-term impact of this course on participants’ ability to translate their skills to clinical environments and the transfer of IPGHC knowledge to the real world. Surveying participants in a decade to assess how IPGHC influenced their trajectory (education, career) and the role of global health in their life would also be beneficial in evaluating the longer impact and effectiveness of the course.

## Conclusion

In conclusion, this study emphasizes the need for interprofessional global health education at the university level. Furthermore, it shows the value of a course that is broad and introductory in nature, exposing students to a variety of topics and perspectives. The results of this study explore the benefits of interprofessional discussion when addressing health in a rapidly changing planet. This is supported by students’ ability to apply knowledge in real-time to the context of the COVID-19 crisis. IPGHC not only highlights the necessity of Global Health curricula but demonstrates how rapidly Global Health learners can apply their knowledge to evolving contexts like what has occurred during the COVID-19 pandemic.

## Electronic supplementary material

Below is the link to the electronic supplementary material.

Supplementary Material 1

## Data Availability

The datasets used and analysed during the current study are not available publicly due to the potential risk of identifying participants based on their responses. Participants did not consent to have their individual responses (raw data) published. Portions of de-identified data are however available from the corresponding author (A.X.N.) upon reasonable request and with permission of the University of McGill Faculty of Medicine and Health Sciences Institutional Review Board.
