# Supplementary material for: Student-led interprofessional global health course: learning impacts during a global crisis
Source: BMC Med Educ. 2023 Mar 16;23:166. doi: 10.1186/s12909-023-04116-4 (PMC10019388; doi:10.1186/s12909-023-04116-4)
Supplement: Supplementary file 1 — Supplementary Material 1 [file 12909_2023_4116_MOESM1_ESM.docx]

**APPENDICES**

**Appendix 1.** Pre-Course Survey IPGHC 2020

*PARTICIPANT DEMOGRAPHICS*

*Please note that the following information is for data collection purposes only.*

*Your name, program, and personal information will never be disclosed, or associated with any publishable data.*

1. Email
   [Valid email address]
2. Participant code
   *(First two letters of last name + last three digits of student number)*
   *For pre- and post-survey matching ONLY.*
   [Short-answer text]
3. Program and Year of study
   [Short-answer text]
4. What is your current understanding of the meaning of global health?
   *Please provide a brief definition to the best of your understanding.*
   [Long-answer text]
5. Do you have previous field work or volunteering experience abroad?
   [Yes]
   [No]
6. If yes, please describe briefly your experience(s).
   *Provide: type of experience (e.g. medical, orphanage, teaching, etc.), length and location*
   [Long-answer text]
7. If yes, please answer the following: Were you ever placed in a situation where you were given responsibility for tasks that you were not trained to do?
   [Yes]
   [No]
8. What are some current skills you believe you possess with regards to Global Health? Skills are related to the "how to" of practice and include practical approaches to fostering reasoning and reflexivity, application of strategies at multiple levels, having an action plan for navigating ethical challenges, etc....
   [Short-answer text]
9. What skills would you like to learn in the course?
   [Short-answer text]
10. Please briefly provide your opinion on the ethical challenges surrounding volunteer and field experience abroad.
    *This question applies to all students, experience or no experience abroad.*
    [Long-answer text]
11. Do you have previous experience working or volunteering with underserved or vulnerable populations at a local level?
    [Yes]
    [No]
12. Please select the rating that best corresponds to your personal viewpoint.
    *1 = strongly disagree, 2 = somewhat disagree, 3 = neutral, 4 = somewhat agree, 5 = strongly agree*

Rows:

| My current program incorporates global health content in its curriculum |
| --- |
| Global health education is important in my professional health care programs |
| Global health education is important in all professional health care programs |
| My professional practice can be influenced by global health knowledge |
| Global health concepts can be applied in a local context |
| Health professionals should do international or remote service work |
| Health professionals have a responsibility to address social determinants of health |
| International electives can help me better apply social determinants of health in a local context |
| My post-secondary institution has a responsibility to expose students to global health opportunities |
| I understand the roles and responsibilities of other healthcare professionals |
| Inter-Professional collaboration is important in addressing global health issues |

Columns:

| 1 | 2 | 3 | 4 | 5 |
| --- | --- | --- | --- | --- |

1. Please rate your knowledge of the following topics.
   *1 = very poor, 2= poor, 3 = fair, 4 = good, 5 = very good*

Rows:

| Social determinants of health |
| --- |
| Global burden of disease |
| Ethics in global health |
| Role of politics and policies in global health |
| Access to healthcare in underserved populations |
| Challenges of healthcare in a low resource setting |
| Cultural sensitivity |
| Canadian Indigenous health |
| Refugee and immigrant health |
| Maternal and child health |
| Sexual violence |
| Systemic racism in healthcare |
| Advocacy and political action |
| Realities of global health fieldwork |
| Healthcare systems |
| Global health and environmental change |
| Role of technology in global health |
| Infectious diseases and Neglected Tropical Diseases |
| Research and development of drugs |
| Nutrition |
| Mental health in the global context |
| Trauma in the global health context |
| Global surgery |

Columns:

| 1 | 2 | 3 | 4 | 5 |
| --- | --- | --- | --- | --- |

1. What is your main motivation for participating in the Interprofessional Global Health Course?
   [Long-answer text]
2. How interested are you in participating in global health work locally or internationally?
   [Not interested]
   [Undecided]
   [Interested]
   [Actively looking]
   [Already involved]

**Appendix 2A.** Post-Course Survey IPGHC 2020

*PARTICIPANT DEMOGRAPHICS*

*Please note that the following information is for data collection purposes only.*

*Your name, program, and personal information will never be disclosed, or associated with any publishable data.*

1. Email
   [Valid email address]
2. Participant code
   *(First two letters of last name + last three digits of student number)
   For pre- and post-survey matching ONLY.*
   [Short-answer text]
3. Program and Year of study
   [Short-answer text]
4. What is your current understanding of the meaning of global health?
   *Please provide a brief definition to the best of your understanding.*
   [Long-answer text]
5. What skills do you feel you learned in the course? Skills are related to the "how to" of practice and include practical approaches to fostering reasoning and reflexivity, application of strategies at multiple levels, having an action plan for navigating ethical challenges, etc....
   [Short-answer text]
6. Please briefly provide your opinion on the ethical challenges surrounding volunteer and field experience abroad.
   *This question applies to all students, experience or no experience abroad. Please elaborate on any new views you may have thought about or learned about during the course.*
   [Long-answer text]
7. Do you have previous experience working or volunteering with underserved or vulnerable populations at a local level?
   [Yes]
   [No]
8. If you answered no to the above question, did the content of the course encourage or motivate you to want to participate in such activities?
   [Yes]
   [No]
9. Please select the rating that best corresponds to your personal viewpoint.
   *1 = strongly disagree, 2 = somewhat disagree, 3 = neutral, 4 = somewhat agree, 5 = strongly agree*

Rows:

| My current program incorporates global health content in its curriculum |
| --- |
| Global health education is important in my professional health care programs |
| Global health education is important in all professional health care programs |
| My professional practice can be influenced by global health knowledge |
| Global health concepts can be applied in a local context |
| Health professionals should do international or remote service work |
| Health professionals have a responsibility to address social determinants of health |
| International electives can help me better apply social determinants of health in a local context |
| My post-secondary institution has a responsibility to expose students to global health opportunities |
| I understand the roles and responsibilities of other healthcare professionals |
| Inter-Professional collaboration is important in addressing global health issues |

Columns:

| 1 | 2 | 3 | 4 | 5 |
| --- | --- | --- | --- | --- |

1. Please rate your knowledge of the following topics.
   *1 = very poor, 2= poor, 3 = fair, 4 = good, 5 = very good*

Rows:

| Social determinants of health |
| --- |
| Global burden of disease |
| Ethics in global health |
| Role of politics and policies in global health |
| Access to healthcare in underserved populations |
| Challenges of healthcare in a low resource setting |
| Cultural sensitivity |
| Canadian Indigenous health |
| Refugee and immigrant health |
| Maternal and child health |
| Sexual violence |
| Systemic racism in healthcare |
| Advocacy and political action |
| Realities of global health fieldwork |
| Healthcare systems |
| Global health and environmental change |
| Role of technology in global health |
| Infectious diseases and Neglected Tropical Diseases |
| Research and development of drugs |
| Nutrition |
| Mental health in the global context |
| Trauma in the global health context |
| Global surgery |

Columns:

| 1 | 2 | 3 | 4 | 5 |
| --- | --- | --- | --- | --- |

1. How interested are you in participating in global health work locally or internationally?
   [Not interested]
   [Undecided]
   [Interested]
   [Actively looking]
   [Already involved]
2. Has there been any change in your desire to participate in such activities?
   [Yes]
   [No]

**Appendix 2B.** Course Evaluation

*Please help us make this course better for your incoming colleagues! While this evaluation is not anonymous, we promise it will not affect anything. It is strictly used to see how we can improve the course for the coming years.*

1. The course was interesting and provided a great introduction to various global health topics.
   [Strongly disagree]
   [Disagree]
   [Neutral]
   [Agree]
   [Strongly Agree]
2. The course was effective in conveying the on-going global health issues around the world.
   [Strongly disagree]
   [Disagree]
   [Neutral]
   [Agree]
   [Strongly Agree]
3. The course content was relevant and was presented in an appropriate amount of depth.
   [Strongly disagree]
   [Disagree]
   [Neutral]
   [Agree]
   [Strongly Agree]
4. The course was well-organized and structured.
   [Strongly disagree]
   [Disagree]
   [Neutral]
   [Agree]
   [Strongly Agree]
5. The course held up to its name as an "Inter-professional Global Health Course" as presented.
   [Strongly disagree]
   [Disagree]
   [Neutral]
   [Agree]
   [Strongly Agree]
6. The course was pleasant and met your expectations.
   [Strongly disagree]
   [Disagree]
   [Neutral]
   [Agree]
   [Strongly Agree]
7. You would recommend the course to a fellow colleague.
   [Strongly disagree]
   [Disagree]
   [Neutral]
   [Agree]
   [Strongly Agree]
8. Please let us know if there is anything specific that you did and/or didn't like? Any suggestions would be appreciated.
   [Long-answer text]

*LECTURER EVALUATION*

*Please rate each lecturer holistically, taking into consideration their enthusiasm, knowledge of topic, lecture style, content, etc. Please rate each professor on a scale of 1-10, 10 being excellent; please provide a rating of 5 if you did not attend the lecture.*

1. Lecture 1 (Jan 7th): Dr. Timothy Evans - Introduction to Global Health

| Terrible | 1 | 2 | 3 | 4 | 5 | 6 | 7 | 8 | 9 | 10 | Brilliant |
| --- | --- | --- | --- | --- | --- | --- | --- | --- | --- | --- | --- |

1. Lecture 2 (Jan 14th): Dr. Yves Bergevin - Reproductive, Maternal, and Child Health: Scaling-up for Sustainable Impact

| Terrible | 1 | 2 | 3 | 4 | 5 | 6 | 7 | 8 | 9 | 10 | Brilliant |
| --- | --- | --- | --- | --- | --- | --- | --- | --- | --- | --- | --- |

1. Lecture 3 (Jan 21th): Glenda Sandy - Indigenous Health

| Terrible | 1 | 2 | 3 | 4 | 5 | 6 | 7 | 8 | 9 | 10 | Brilliant |
| --- | --- | --- | --- | --- | --- | --- | --- | --- | --- | --- | --- |

1. Lecture 4 (Jan 28th): Dr. Paul Allison - Oral Health as Global Health (Lancet Report)

| Terrible | 1 | 2 | 3 | 4 | 5 | 6 | 7 | 8 | 9 | 10 | Brilliant |
| --- | --- | --- | --- | --- | --- | --- | --- | --- | --- | --- | --- |

1. Lecture 5 (Feb 4th): Dr. John Pringle - Contemporary Issues in Humanitarian Action

| Terrible | 1 | 2 | 3 | 4 | 5 | 6 | 7 | 8 | 9 | 10 | Brilliant |
| --- | --- | --- | --- | --- | --- | --- | --- | --- | --- | --- | --- |

1. Lecture 6 (Feb 11th): Dr. Srividya Iyer - Mental Health in Global Health

| Terrible | 1 | 2 | 3 | 4 | 5 | 6 | 7 | 8 | 9 | 10 | Brilliant |
| --- | --- | --- | --- | --- | --- | --- | --- | --- | --- | --- | --- |

1. Lecture 7 (Feb 18th): Dr. Jena Webb - Ecosystems Approach to Health & Climate

| Terrible | 1 | 2 | 3 | 4 | 5 | 6 | 7 | 8 | 9 | 10 | Brilliant |
| --- | --- | --- | --- | --- | --- | --- | --- | --- | --- | --- | --- |

1. Lecture 8 (Feb 25th): Dr. Raphael Lencucha - Global Health Policy & Politics

| Terrible | 1 | 2 | 3 | 4 | 5 | 6 | 7 | 8 | 9 | 10 | Brilliant |
| --- | --- | --- | --- | --- | --- | --- | --- | --- | --- | --- | --- |

1. Lecture 9 (recorded lecture): Dr. Daniele Lantagne - Environmental Health

| Terrible | 1 | 2 | 3 | 4 | 5 | 6 | 7 | 8 | 9 | 10 | Brilliant |
| --- | --- | --- | --- | --- | --- | --- | --- | --- | --- | --- | --- |
